# Albuminuria and Bright's Disease

**Published:** 1900-06

**Authors:** 


**Albuminuria and Bright's Disease.** By NESTOR TIRARD, M.D. Pp. 362. London: Smith, Elder, & Co. 1899.

This is a detailed and careful exposition of the majority of kidney diseases, studiously moderate in tone, and the fruit of matured study and wide experience. It commences with an apology for the title, which may be thought wanting in scientific precision, but sufficiently expresses the aim of the author. He, like Sir George Johnson, looks upon Bright's class of kidney troubles as secondary to a general constitutional disease, while albuminuria, though so frequent a symptom, is found also in a large number of diseases where the kidney does not undergo any organic change. Thus besides extra-renal albuminuria and that seen in Bright's diseases, he recognises that due to mechanical changes, *e.g.* in heart disease, and to changes in the blood from fevers and toxins, as well as purely functional and cyclic forms. It is not very clear why he does not include most forms of Bright's disease under the results of intoxications, but apparently he would say the poison in them is an unknown one, if it actually exists; and on the other hand, the toxic albuminuria of fevers differs in that it does not usually lead to structural changes of a permanent character. The puerperal form is recognised as of renal origin, though sometimes unassociated with structural changes, and passing away with remarkable rapidity. In this, as in other subjects, we find various theories given, but the author does not regard the question as ripe for dogmatic statements. The tests for albumin recommended are the nitric acid, cold and after boiling, trichloracetic and picric acids. The fallacy from mucin or nucleo-albumin with the last named is not mentioned, and we wish that the diagnosis of this body from serum albumin by other tests had been given more fully. Dr. Samuel West discussed the matter very ably in his recent lectures, and pointed out the sources of error.

It would have been well to add a definite rule for deciding whether the albumin in urine containing pus or blood is due solely to these substances or not. By counting the leucocytes present we can, according to some authorities, estimate the albumin due to these conditions and compare it with the total amount present. An interesting discussion is to be found on the much debated subject of prognosis in functional albuminuria, and careful precautions are enjoined lest a true renal disease should be overlooked. The author, after a long experience in insurance work, is of opinion that real cases of this class very rarely give rise to permanent renal trouble.

On the whole, the work is full of information, though a little wanting in definiteness. The style is clear, but the same subject is discussed in different places, so that a good deal of repetition ensues, and it is not always easy to find the author's final views. We have only noticed a single misprint, "tanna-form," for "tannoform," a word which has as yet hardly attained to settled use (page 259). The index is a valuable addition to the book, and the beauty of the coloured plates of retinal changes, after drawings by Stanford Morton, will be at once recognised.

